# Operating with Data - Statistics for the Cardiovascular Surgeon: Part III. Comparing Groups

**DOI:** 10.21470/1678-9741-2018-0378

**Published:** 2018

**Authors:** Gabriel Romero Liguori, Luiz Felipe Pinho Moreira

**Affiliations:** 1 Laboratório de Cirurgia Cardiovascular e Fisiopatologia da Circulação (LIM-11), Instituto do Coração (InCor), Hospital das Clínicas HCFMUSP, Faculdade de Medicina, Universidade de São Paulo, São Paulo, SP, Brazil.

In the previous issues of the Brazilian Journal of Cardiovascular Surgery (BJCVS) we discussed, first, the fundamental concepts required for understanding biostatistics^[1]^ and, then, how to evidence associations and assess risk^[2]^. In this third part of the editorial series entitled "Operating with Data - Statistics for the Cardiovascular Surgeon", we will examine the methods for comparing groups.

## Comparing What?

Here, again, it is important to clarify what we define as "comparing groups". One could state that, in our last editorial^[2]^, we were also comparing groups. Indeed, we were comparing the occurrence of determined events among two or more groups. However, since both variables were qualitative (or categorical), we defined those cases as an analysis of association. Now, we are referring to the comparison of quantitative (or numerical) variables in two or more groups. In these cases, the object of analysis is not the frequency of the events, as before, but the central value of a quantitative variable. Scientifically speaking, the methods we will present in this editorial are valid for those cases in which the independent variable is qualitative *i.e*. groups and the independent variable is quantitative.

**Fig. 1:**
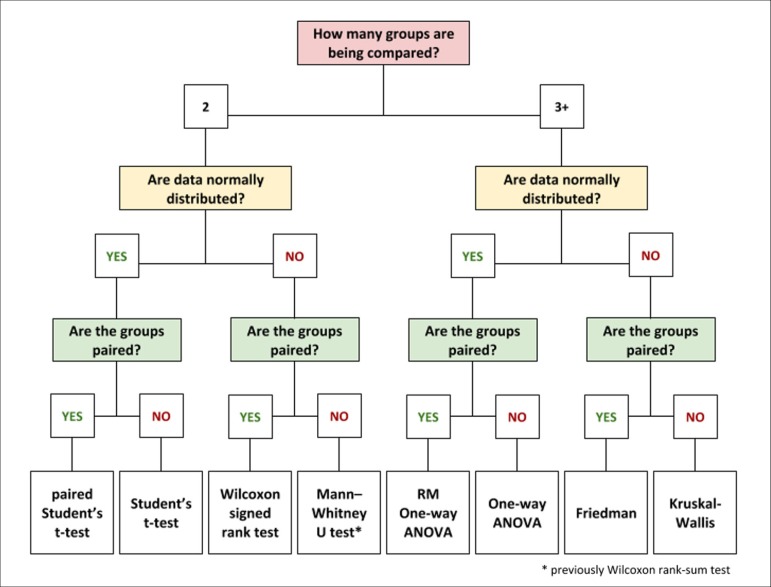
Decision flowchart for group comparison.

Luckily, tests for comparing groups, as defined above, are probably the simplest statistical tests to understand, if you are not willing to go deep in the mathematical side of them, which is the case for us. In fact, the whole editorial could be summarized in three simple questions (Figure 1): 1) How many groups are being compared?; 2) Are the data normally distributed?; and 3) Are the groups paired?. The concepts required to answer questions 2 and 3 *i.e*. data distribution and pairing are fully explained in our first editorial^[1]^. Still, it is important to understand what is behind each of these different tests and, thus, comprehend why they are the choice for each of these different situations.

## Comparison Tests for Two Groups

If you are working with only two groups, let's say intervention and control, somehow you will be using a t-test. The t-distribution (and consequent t-test) was first proposed in 1908 by William Gosset^[3]^, a chemist from Guinness brewery who could not publish his findings under his own name and, thus, did it under the pseudonym of "Student", reason why the most used t-test is named Student's t-test. The idea of all the t-tests is the same: to answer if the observed difference is larger than we should expect from random inconstancy.

**Fig. 2:**
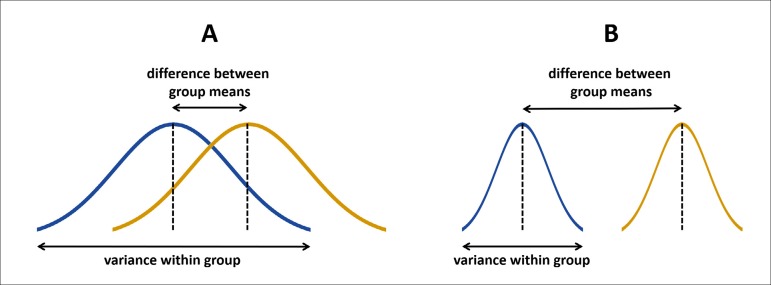
Graphical representation of the rationale behind the Student's t-test. A) Groups not significantly different. B) Groups significantly different.

For that, the t-test calculates the ratio of the difference between group means and the variance within the groups (Figure 2) to define a t-value. Thus, if the difference between the means is small and the variance within the groups is large, the t-value is low (Figure 2A). Oppositely, if the difference is large and the variance is small, then the t-value is high (Figure 2B). The higher is the t-value, the most significant is the difference. Using this t-value and the degrees of freedom of the sample (which is related to the number of observations), the t-test calculates the *P*-value for that difference. In the case of paired data, you can use the paired version of the Student's t-test. The details of the test's mathematical formula do not belong to the scope of this editorial but can be easily found online. The Student's t-test, however, is somehow limited because it assumes the data is normally distributed and the standard deviation is the same for both groups. When data is not normally distributed, however, other approaches are necessary to test the difference between the groups.

**Fig. 3:**
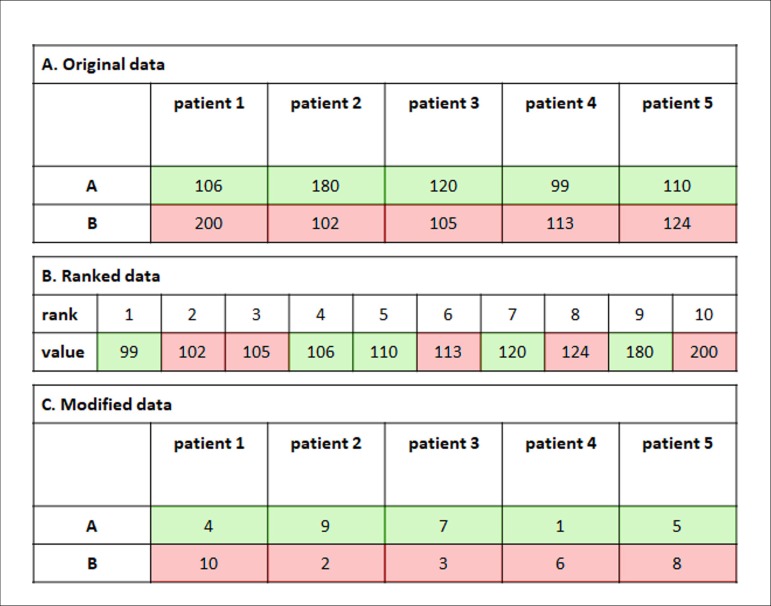
Statistics based on ranks. A) Original data. B) Ranked data. C. Modified data.

Frank Wilcoxon, in 1945, proposed a modification to the Student's t-test that allowed Gosset's calculations to be used for non-normal distributions^[4]^. Briefly, the proposed approach was to put all the data from both groups together and organize it in an ascending manner, so that each value would, now, possess a position within an ordered set of values. This position was called rank and this rank was used for calculating the t-test, instead of the actual value. This test was called Wilcoxon rank-sum test (do not confound with the Wilcoxon signed rank test). To illustrate this concept, let's imagine we have two groups of five patients, A and B, undergoing cardiopulmonary bypass (CPB). The time under CPB was registered and tabulated as in Figure 3A. These values were, then, brought together and reorganized in an ordered sequence, as in Figure 3B, so that for each value it was assigned a rank. The rank, then, substitutes the original value in the original table, originating a new table, as in Figure 3C. The values in Figure 3C are the ones which will be used to calculate the Student's t-test. Naturally, you do not need to perform all these steps when running a Wilcoxon rank-sum test, the statistics software does it all automatically, but it is interesting to understand how the test is performed so that you can better comprehend its applications. The Wilcoxon rank-sum test, however, also presented limitations, one of them being the fact that it could only be used for groups with equal numbers of subjects. In order to solve this issue, two statisticians, Mann and Whitney, proposed, in 1947, a modification to the formula of the Wilcoxon rank-sum test so that groups of different sizes could be evaluated^[5]^, originating the Mann-Whitney U test, also referred as Mann-Whitney-Wilcoxon (MWW) test. Still, the whole concept behind this test is also the ranking of the original values and further calculations with the rank values.

Still, none of the tests, neither the Wilcoxon rank-sum test nor the Mann-Whitney U test were designed to evaluate paired data. To do this, Wilcoxon proposed, in the same publication he proposed the rank-sum test, another test, specific for paired data^[4]^. This test is what is called the Wilcoxon signed rank test. Because the groups of paired data will always have the same size, this test did not require the modifications proposed by Mann and Whitney and thus continue to be the choice for the comparison of two paired groups with non-normal distribution. 

## Comparison Tests For Three or More Groups

When working with three or more groups, like comparing treatments A, B, and C, another type of statistical test must be used, the so-called analysis of variance (ANOVA). Maybe some of the readers are asking themselves why not to perform multiple t-tests so to compare the multiple groups in separate analyses. The answer is actually very simple: by using this approach *i.e*. multiple t-tests, the researcher would be increasing the type I error. This means he or she would be rejecting the null hypothesis when it is, in fact, true or, putting it in simpler words, affirm there is a difference among the groups which actually does not exist. Suppose you have five different groups: A, B, C, D, and E. If you would use only t-tests, you would have to perform ten different t-tests (A *vs*. B; A *vs*. C; A *vs*. D; A *vs*. E; B *vs*. C; B *vs*. D; B *vs*. E; C *vs*. D; C *vs*. E; and D *vs*. E). In each of these tests, you assume an acceptable error of 5% *i.e*. *P*=0.05. Thus, after performing all the tests, your final error is up to 50%, meaning that if you find a difference between two of those five groups by running multiple t-tests, the possibility of that difference being due to chance is 50%! To solve this issue, the analysis of variance was created.

**Fig. 4:**
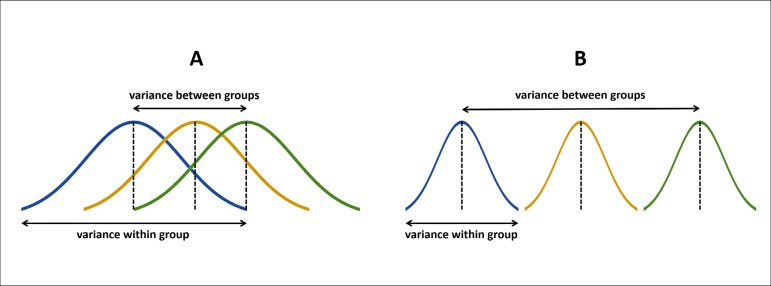
Graphical representation of the rationale behind the analysis of variance (ANOVA). A) Groups not significantly different. B) Groups significantly different.

To compare several groups at once keeping a fixed type I error, the analysis of variance calculates two types of variance *i.e*. the spread between numbers in a data set. The first is the variance within each group, what is done by calculating the variance between each observation in a group and this group mean. Then, it is calculated the variance between the groups, which, in turn, is done by calculating the variance between each group mean and the overall mean (the mean of all values in all groups). Finally, the ratio between the variance and the within variance (b/w) is calculated. If the ratio is large, it means the groups differ, if the ratio is low, it means the groups do not differ. Figure 4 illustrates very well this rationale. When the variance between groups is smaller than the variance within the group (Figure 4A), there is probably no difference among these groups. On the other hand, when the variance between groups is larger than the variance within the group (Figure 4B), there is probably a difference among them. To calculate if this ratio *i.e*. the difference among groups is statistically significant it is used the F-test. Similarly, to the t-test, several variables are used in this calculation and the mathematical details of this formula will not be covered by this editorial. Besides understanding the rationale behind the analysis of variance, it is also important to recognize that, although this test can inform if there is at least one group that differs from the others, it cannot state which is the different group and what is size or direction of this difference. For that, *post-hoc* tests are necessary, and we will discuss them later in this editorial.

The analysis of variance above described is what is traditionally called One-way ANOVA and is valid for normally distributed data and non-paired groups. Other types of analysis of variance, however, can also be performed in cases where data is not normally distributed and/or if the groups are paired. First, for the cases in which data is normally distributed, but paired, the test of choice will be the Repeated Measures (RM) One-way ANOVA. As any test for paired samples, RM One-way ANOVA will consider the variations within each subject when making the previously explained calculations. The second variation of the One-way ANOVA is the so-called Kruskal-Wallis test, also known as One-way ANOVA on Ranks, which was designed by these two statisticians, William Kruskal and W. Allen Wallis, for variables which are both not paired and not normally distributed^[6]^. The Kruskal-Wallis test is a derivation of the Mann-Whitney U test and, thus, does not assume a normal distribution of the data and uses its ranked values for calculations. Finally, the third variation of the One-way ANOVA is valid for variables which are paired but not normally distributed. This test was developed by the Nobel laureate Milton Friedman^[7]^ and, for this reason, is called the Friedman test. To think this test in a simple way, it could be explained as a combination of the two previous tests *i.e*. it is a ranked test for repeated measures.

## *Post-hoc* tests

As commented above, all the previously described tests only inform if there is at least one group that differs from the others, but do not state which is the different group and what is size or direction of this difference. For dissecting these differences, a *post-hoc* test is necessary. *Post-hoc* tests should only be performed after a statistically significant difference was found in the analysis of variance. These tests use different means to determine what is the different group among all others. In fact, the *post-hoc* test can be performed in three different ways: 1) comparing all groups against each other (all pairwise comparison); 2) comparing specific pairs of interest (specific pairwise comparison); or 3) comparing all treatment groups against one control group. Not all *post-hoc* tests can be used for any of these three situations. Also, not all *post-hoc* tests can be used after any parametric or non-parametric analysis of variance. Table 1 summarizes when each of the most used *post-ho*c tests should be used. Another important observation is that each *post-hoc* tests are more or less prone to type I or type II errors (for definitions, check our first editorial (1)) so that they are more liberal or more conservative in regard to accepting false-positives in order to not risk false-negatives. Table 1 also list the limitations of each test, such as the type of error each test is more prone to incur and other statistical pitfalls. Other *post-hoc* tests not described in Table 1 exist, but this editorial does not intend to cover all of them.

**Table 1:** *Post-hoc* tests.

Test	ANOVA	Comparison	Requirements and limitations
Fisher's LSD	Parametric (One-way ANOVA or RM One-way ANOVA)	All pairwise comparisons, specific pairwise comparisons and compare treatments with a control	Prone to type I error
Holm-Sidak	Parametric (One-way ANOVA or RM One-way ANOVA)	All pairwise comparisons, specific pairwise comparisons and compare treatments with a control	Prone to type II error and does not give confidence interval (only significance)
Bonferroni	Parametric (One-way ANOVA or RM One-way ANOVA)	All pairwise comparisons, specific pairwise comparisons and compare treatments with a control	Prone to type II error
Tukey-Kramer	Parametric (One-way ANOVA or RM One-way ANOVA)	Only for all pairwise comparisons	Prone to type I error (less than Fisher's LSD)
Newman-Keuls	Parametric (One-way ANOVA or RM One-way ANOVA)	Only for all pairwise comparisons	Require an equal number of subjects in all groups; prone to type II error; and does not give confidence interval (only significance)
Dunnet	Parametric (One-way ANOVA or RM One-way ANOVA)	Only when comparing treatments with a control	Prone to type II error
Dunn's	Non-parametric (Kruskal- Wallis or Friedman)	All pairwise comparisons, specific pairwise comparisons and compare treatments with a control	Prone to type II error and does not give confidence interval (only significance)

## The Two-way ANOVA

Finally, it is important to point to the existence of a Two-way ANOVA. The Two-way ANOVA is a type of analysis of variance for when you have two independent variables being analyzed at the same time. One example could be the evaluation of cardiac function after 30, 120 and 180 days after patients were submitted to two different approaches, A and B, of myocardial revascularization. The first variable is the intervention, which could be A or B. The second variable is the time at evaluation, which could be 30, 120, or 180 days. Performing a Two-way ANOVA can lead to three different conclusions: 1) if there are differences due to the intervention group; 2) if there are differences due to the time point; and 3) if there are differences due to a combination of intervention group and timepoint. This combination is called interaction and, if significant, means that differences found in one of the independent variables could also be partially attributed to the other, making it difficult to determine what is, in fact, the main variable responsible for the observed effect. Two-way ANOVA can also be followed by *post-hoc* tests, many of which are the same used for One-way ANOVA.
